# Periodontitis and Depressive Disorders: The Effects of Antidepressant Drugs on the Periodontium in Clinical and Preclinical Models: A Narrative Review

**DOI:** 10.3390/jcm13154524

**Published:** 2024-08-02

**Authors:** Damiano Taccardi, Alessandro Chiesa, Carolina Maiorani, Alessia Pardo, Giorgio Lombardo, Andrea Scribante, Silvia Sabatini, Andrea Butera

**Affiliations:** 1 Unit of Dental Hygiene, Section of Dentistry, Department of Clinical, Surgical, Diagnostic and Pediatric Sciences, University of Pavia, 27100 Pavia, Italy; damiano.taccardi01@universitadipavia.it (D.T.); alessandro.chiesa@hotmail.it (A.C.); andrea.scribante@unipv.it (A.S.); andrea.butera@unipv.it (A.B.); 2 Section of Oral and Maxillofacial Surgery, Department of Surgical Sciences, Dentistry, Gynecology and Pediatrics, University of Verona, 37124 Verona, Italy; giorgio.lombardo@univr.it; 3 Unit of Orthodontics and Pediatric Dentistry, Section of Dentistry, Department of Clinical, Surgical, Diagnostic and Pediatric Sciences, University of Pavia, 27100 Pavia, Italy; 4 Department of Surgical, Medical, Dental and Morphological Sciences, University of Modena and Reggio Emilia, 41124 Modena, Italy; silviasabatini01@gmail.com

**Keywords:** antidepressant, periodontitis, inflammation, neuroinflammation, systematic diseases

## Abstract

**Background/Objectives**: Several psychological conditions, including stress and depression, can adversely affect oral health; in fact, antidepressants, commonly used to treat depressive disorders, may have conflicting effects on the periodontal status of individuals. The aim of this review was to determine the effects of antidepressants on the periodontium. **Methods:** A literature search was conducted using electronic databases, Pubmed/MEDLINE, Cochrane Library, focusing on the use of antidepressants and their effects on periodontal health in animals or humans. **Results**: Seventeen articles have been included with the use of amitriptyline (two studies), desipramine (one study), imipramine (two studies), desvenlafaxine (one study), fluoxetine (six studies), venlafaxine (three studies) and tianeptine (two studies). One study evaluated several categories of antidepressants, such as selective serotonin reuptake inhibitors (SSRI), serotonin-norepinephrine reuptake inhibitors (SNRI), tricyclic, atypical and monoamine oxidase inhibitors (MAO). Most trials showed improvements in periodontal health, especially with fluoxetine, but also with imipramine, desipramine, desvenlafaxine and tianeptine; on the contrary, worsening of clinical periodontal indices and increased loss of alveolar bone were reported with venlafaxine. **Conclusions**: This review suggests that in the presence of comorbidity between periodontitis and depression, pharmacological treatment with SNRIs, SSRIs and mixed antidepressants is associated with improvement in periodontal parameters, except for venlafaxine. Healthcare professionals (especially oral and mental health professionals) should investigate proper adherence to medication therapy in patients with a history of periodontitis and depression. Further clinical trials are needed to confirm these results.

## 1. Introduction

Periodontal disease is a problem that affects the worldwide population: global cases of severe periodontitis were reported to be approximately 1.1 billion in 2019, an increase of 99% from 1990 to 2019, and this is attributable to population growth and increased ageing [1]. It affects almost 40% of the world’s population [2].

Periodontitis is a chronic inflammatory disease of multifactorial etiology associated with dysbiotic plaque biofilms that progressively weaken and destroy the oral tissues supporting the tooth within the alveolar bone [3,4,5]. Periodontitis can be successfully treated with various therapies, such as mechanical or manual instrumentation, which aim to remove the bacterial biofilm, reduce bleeding on probing and promote healing of the oral tissues [6,7,8,9]. 

Recent evidence suggests that there may be several mechanisms linking poor oral health to mental and affective disorders; poor oral hygiene and periodontal disease may lead to low-grade chronic systemic inflammation, which is considered a risk factor for neuropsychiatric disorders [10,11,12,13]. It is well known that oral bacteria can enter the bloodstream by damaging the gums, and if the blood–brain barrier is weakened, they can also enter the brain via pro-inflammatory mediators; periodontal disease can also indirectly affect the central nervous system [14,15,16,17]. People with anxiety or depression have been found to have a higher incidence of tooth decay and loss, and people with bipolar disorder have been found to have a higher risk of periodontitis: contributing factors include poor oral hygiene and diet, tobacco use, alcohol and psychostimulants [18,19,20,21].

Affective disorders are psychopathological disorders characterized by a change in the individual’s general activity, with alterations at an emotional level, mood and motivation, which manifest themselves in a reduction in functioning at a personal, work and social situations [22].

The DSM-5, a statistical and diagnostic manual of mental disorders, differentiates mood disorders into two distinct categories: depressive disorders and bipolar disorders. Major depressive disorder, also called endogenous depression, manifests itself mainly with a depressed mood, lack of motivation and vital energy, poor concentration ability, guilt, insomnia or hypersomnia, psychomotor slowing or agitation, apathy or loss of interest, and finally, suicidal ideation or attempts [22,23,24]. Unlike depressive disorders, which are characterised by a single polarity (i.e., the mood disorder is manifested only in the depressive variant), in bipolar disorders we observe the presence of manic or hypomanic episodes alternating with depressive episodes. In mania or hypomania, the mood is defined as “expanded”, elevated, euphoric: a patient before a manic episode may show logorrhea, acceleration of thought content, reduced need for sleep, psychomotor activation, up to and including excessive expenditure, and uninhibited behaviour [24,25]. This disorder is manifested by a sense of grandeur, decreased need for sleep, logorrhea, accelerated thinking, other distractibility, a significant increase in daily activities and excess risky activities. The diagnostic criteria for major depressive disorder are met when five of the above symptoms occur simultaneously for two weeks and cause clinically significant distress to the individual; for bipolar disorder, however, at least three of the above symptoms may occur for at least four days [25,26].

Currently, depression is the most common mental illness in the world: the number of cases from 1990 to the present has increased by 49.86% (from 172 to approximately 258 million in 2017), with major depressive disorder reported as the most widespread in the population [27]. Furthermore, the World Health Organization (WHO) indicates that this specific depressive disorder will become the leading cause of disability worldwide by 2030 [28]. Worldwide, the incidence of mood disorders is second only to that of anxiety disorders, affecting a large proportion of the world’s population. In Italy, according to the European Study on the Epidemiology of Mental Disorders, the lifetime prevalence of major depression and dysthymia is 11.2%. Thus, it is relatively common to develop a mood disorder over the course of a lifetime. It is also likely that some changes in Western society will favour a greater prevalence of these disorders [22,23,24,29].

Periodontal disease and depressive disorders have several risk factors in common, including smoking and lifestyle. Both are highly prevalent pathologies in their respective areas of concern: having a mental disorder results in reduced self-care and a potential increase in periodontal disease [30,31,32,33].

In a preclinical in vivo study, periodontitis and subsequent experimental depression were induced in a group of rats: the group in which both periodontitis and depression were induced (periodontitis was induced by inoculating P. gingivalisATCC W83 K1 and F. nucleatum DMSZ 20482 by oral gavage). The rat group with Nucleatum DMSZ 20482 by oral gavage, while depression was induced by introducing a series of different stressors that changed daily (two stressors/day)), showed significantly greater alveolar bone loss at the level of the bifurcation than the non-depressed groups with induced periodontitis. *Fusobacterium nucleatum* has been found in the frontal cortex of mice with periodontally induced depression [34]. 

Starting from preclinical studies on animal models, other studies have tried to investigate the associations between these pathologies in subjects with periodontitis compared to healthy individuals. A study comparing approximately 50,000 healthy subjects vs. 12,000 with periodontitis demonstrated that subjects with periodontitis were more likely to develop depression within 10 years of follow-up, indicating periodontitis as a risk factor for depression later, regardless of other associated comorbidities such as sex and age [35]; not only could periodontal disease be a risk factor for affective disorders: depression could also be a risk factor for periodontal disease, indicating a two-way link [36]. 

A recent meta-analysis, published in the *Journal of Clinical Periodontology* in 2021, indicated the plausibility of a bidirectional depression–periodontitis association, dividing the possible mechanisms involved into three aspects: behavioural, biological/physiological and bacteriological aspects [37].

Depressed subjects tend to engage in riskier behaviours, which can worsen oral health and predispose them to periodontitis [33,34,35,36,37,38]. Periodontitis is one of the main causes of edentulism in the population, which can compromise the contour and aesthetics of the face [39]. Periodontal disease significantly affects the patient’s quality of life and masticatory function, as well as worsens self-confidence: all of these factors can predispose periodontal patients to a significant worsening of mood, with an increased risk of depression [40,41,42,43,44,45].

Individuals with depression report increased levels of certain inflammatory production factors, including interleukin-6 (IL-6), tumour necrosis factor (TNF)-alpha, interleukink-10 (IL-10), as well as several soluble receptors and interleukin antagonists and cytokines, such as the soluble IL-2 receptor, IL-13 and IL-1 receptor antagonist [46,47,48]. From here, the hypothesis of “inflammatory depression” was born, a real subtype of depression, which identifies mechanisms that act as catalysts/amplifiers of responses: bad eating habits, stress and sedentary lifestyle [49]. In contrast, among the possible protective factors against the development of depression, probiotics have been proposed and studied as adjuvants in the non-surgical treatment of periodontitis [49,50,51].

Therefore, individuals with depression and periodontitis appear to share an alteration in the production of various pro-inflammatory factors (IL-1, IL-6, TNF-α), which, on the one hand, increases the individual’s systemic inflammatory load [52] and, on the other hand, increases neuroinflammation [53,54]. Cortisol-related stress is also associated with periodontitis [55]. In fact, cortisol could be differentiated into different forms of periodontitis: subjects with aggressive periodontitis had higher cortisol levels than subjects with chronic periodontitis [56]. Cortisol, resulting from the incorrect functioning of the hypothalamic–pituitary–adrenal (HPA) axis, induces endocrine and cerebral changes, increasing vulnerability to the development of depressive pathologies. Therefore, by increasing its serum production, it can cause the reduction in cellular trophism of neurons and the inhibition of neurogenesis mechanism [57], all of which seem to significantly predispose subjects to mental pathology [58]. Similarly, “inflammatory depression” induces a functional alteration of the HPA axis with increased secretion of pro-inflammatory cytokines such as IL-6 and TNF-α; the latter are in turn associated with the development of periodontitis [37,47,52,54,59]. Therefore, depression and periodontitis share a potential bidirectionality in the etiology and maintenance of pathology [60]. 

Virulence factors of some periodontal disease pathogens can overcome the blood–brain barrier and negatively influence the physiology of the brain via glial cells, which are mediators of various neural signals [61,62]. 

Antidepressants are widely used in the population, particularly in the periodontal population: subjects with periodontitis are more likely to take antidepressant drugs when compared to healthy patients [63]. Although the various anti-inflammatory functions of these drugs are well known in the literature, acting also at the level of systemic inflammation and enhancing the individual’s immune response [64,65,66], the literature seems to be divided on their effects at the periodontal level, except for one study where it was found that the use of antidepressants could protect against periodontal disease, noting a lower loss of clinical attachment; the explanation of this effect is unclear [67]. However, other studies in the literature mention some factors at the oral level, such as bacterial infections and accumulation of bacterial biofilm, which favour the development of periodontal disease [68,69].

Therefore, the aim of this study was to determine the periodontal effects of the antidepressant drugs most commonly used to treat different forms of depression, as there are few studies focused on researching the periodontal effects of these drugs.

## 2. Materials and Methods

### 2.1. Hypothesis

Are there any periodontal effects of antidepressant drugs? What are the effects of antidepressants at the periodontal level?

### 2.2. Eligibility Criteria

The following inclusion criteria guided the analysis of the studies:

Study type. Clinical and preclinical studies in patients or animals, i.e., randomised, blind/double-blind clinical trials, case–control, cross-sectional, cohort and observational studies and reviews;

Type of participants. Patients or animals with experimentally induced periodontitis and/or depression;

Type of interventions. Pharmacological treatments for all clinical and subclinical forms of depression. Changes in parameters of periodontal inflammation and diagnosis of periodontitis; changes in laboratory and genetic study variables (serum biomarker titration and gene expression tests);

Type of results. Determination of positive and negative effects of antidepressants at the periodontal level.

Only studies that met all the inclusion criteria were included. However, the following exclusion criteria were considered: articles published before 2005, in vitro studies, studies evaluating inflammatory parameters in oral pathologies other than periodontal disease (because the focus of this review is periodontal disease), studies in which the comparator molecule is used to treat pathologies associated with depression, such as anxiety disorders (benzodiazepines and beta-blockers), and articles on peri-implantitis and implant loss.

### 2.3. Search Strategy

The population, intervention, comparison, outcome (PICO) model was used to perform this narrative review through research of studies identified in electronic databases, Pubmed/MEDLINE and the Cochrane Library. Initially, all study abstracts were taken into consideration, and all studies that met the inclusion criteria and evaluated changes in periodontal parameters in clinical or pre-clinical models using antidepressant drugs were reviewed and analysed.

We performed the search using the following keywords: ‘’antidepressant and periodontitis”; “antidepressant and periodontal disease”; “antidepressant and periodontal health”; “antidepressant and periodontal status”; and “antidepressant and periodontal inflammation”.

### 2.4. Screening and Selection of Articles

The electronic search yielded 130 results, which were examined to determine whether the inclusion and exclusion criteria were met; all duplicates emerging from the different searches were removed (one authors).

In the first phase, the results (abstracts) were filtered based on the use of pharmacological treatments for all clinical and subclinical forms of depression; all those that did not evaluate changes in the parameters of periodontal inflammation or met the eligibility criteria were discarded. Authors (all) continued with the reading of the articles included.

After reading the selected articles, all those that had not reported the results required by the criteria for inclusion of this review, or which evaluated other oral epidemiological factors and indices other than periodontal health were removed (three authors).

### 2.5. Risk of Bias and Results

One author was involved in the analysis of the articles included and evaluated the results according to the PICO model. Two other authors were commissioned to evaluate the quality of the included studies, following the full reading of the articles [69].

## 3. Results

The main results of these studies are shown in Table 1.

The electronic search yielded 130 results, and 17 articles met the eligibility criteria and were included in this narrative review.

The studies included in the review evaluated the use of several antidepressants, such as amitriptyline (two studies) [69,70], desipramine (one study) [72], imipramine (two studies) [73,74], desvenlafaxine (one study) [75], fluoxetine (six studies) [67,68,77,78,79,80], venlafaxine (three studies) [67,68,76] and tianeptine (two studies) [81,82]. One study evaluated several categories of antidepressants, such as selective serotonin reuptake inhibitors (SSRI), serotonin-norepinephrine reuptake inhibitors (SNRI), tricyclic, atypical and monoamine oxidase inhibitors (MAO) [66]; the only included review investigated the effects of fluoxetine, venlafaxine and tianeptine [83].

The antidepressant drug that seems to make periodontal health worse is venlafaxine (in all studies that have analyzed its effects), with an increase in clinical indices such as gingival index, periodontal pocket depth, clinical attachment loss (*p* < 0.05) [67,68] and inflammation and bone loss (*p* < 0.001) [76].

For the two amitriptyline studies, there are two different results: in one study (RCT) where amitriptyline gel or mouthwash was used, there was an improvement in periodontal health and a reduction in the epidemiological indices (*p* < 0.001) analysed (probing depth, attachment level, tooth mobility, plaque index, gingival index and bleeding on probing) [70], while in the second study (in rats), when amitriptyline was administered as a tablet for 4 weeks, a deterioration in periodontal health was noticed, with reduced bone mineral density [71]. Discordant results also emerge from the studies that analysed the effects of fluoxetine: it reduced bone loss and inflammatory parameters, with noted improvements in gingival index, sulcus bleeding index, bleeding on probing, probing depth, attachment level [77,78,79]; however, other studies have shown a worsening of the epidemiological indices related to periodontal disease [67,68] and a decrease in periodontal cells, although no structural periodontal changes in the analysis were noted [80].

For imipramine, desipramine, desvenlafaxine and tianeptine, there were improvements in periodontal status in all studies.

Table 2 shows a summary of the antidepressant used and its effect on the periodontium.

### Risk of Bias of Studies Included

The evaluation of blinding, randomisation, allocation concealment, outcome data and outcome recording were carried out to assess the bias risk of this review. According to the variable taken into consideration, a green symbol was assigned where the information was complete and accurate; a yellow symbol was allocated where information was missing; and a red symbol was assigned where the information did not meet the requirements. This review has low risk of bias.

Table 3 shows the risk of bias in the main articles examined.

## 4. Discussion

Recent studies [32,84] have suggested a strong link between oral health and systemic diseases, one of which is mental health with depression.

The link between periodontal disease and depression can be explained by behavioural, biological and bacteriological factors.

Periodontitis is an inflammatory disease, and inflammatory factors such as IL-1, IL-6 and TNF-α are directly associated with periodontitis [85]. Some of these biomarkers appear to play a central role in regulating the inflammatory process of the immune response and are identified based on the bone destruction that characterises the destructive phase of the disease [86,87]. In particular, IL-1 and TNF are directly involved in osteoclastic resorption [88] by inhibiting osteoblast activity and promoting the release of CSF-1, also known as macrophage colony-stimulating factor [89]. However, a secondary group of cytokines (such as IL-10) have antagonistic and periodontal protective effects [90]. TNF is also involved in the pathogenesis of periodontitis. In fact, high levels of this cytokine upregulate the expression of RANKL in osteoblasts, T cells and gingival epithelial cells; therefore, it has been hypothesised that it may be associated with the early stage of periodontitis by altering the oral mucosal barrier [91]. These inflammatory factors have also been found in people with depression. In summary, known pro-inflammatory cytokines of the IL-1, IL-6 and TNF families are secreted by periodontal cells and host immune cells after pathological stimulation, which activate and recruit specific immune cell subsets that cause direct tissue damage. Thus, T cells and B cells differentiate into mature T cells or plasma cells under the influence of specific cytokines and further activate or promote other effector cells, such as osteoclasts and neutrophils, which exert pro-inflammatory or anti-inflammatory effects [86,87,88,89,92].

In addition to biological factors, there are virological aspects in common between individuals with periodontitis and depression: *Fusobacterium nucleatum* has been identified in the brain tissue of animals with induced periodontitis, suggesting that this may be associated with HPA dysregulation and elevated corticosterone levels [34]. *Porphyromonas gingivalis*, a major pathogen of periodontitis, inoculated into preclinical models every other day for 4 weeks, induced depression-like behaviours [93]. This is followed by changes in the levels of neurotrophic receptors (in rats with both periodontitis and depression) [34]. Also, the presence of stress hormones catecholamine, dopamine and cortisol increased proliferation and growth of *Tannerella forsythia* and *Fusobacterium nucleatum* [94]. Therefore, stress-related adrenal hormones, such as catecholamines, can modulate the growth of some bacterial species, including *Eikenella corrodens, Tannerella forsythia* and *Fusobacterium nucleatum* [94,95], and drive the transition towards dysbiosis [32], a key element in the pathogenesis of periodontal disease [3].

Moreover, there are certain negative habits common to both diseases. Poor oral hygiene, smoking, alcohol consumption and poor or unregulated nutrition remain influential factors in both cases.

The articles analysed in the following review included 17 in vivo studies, which complied with the inclusion criteria, to understand the effects of antidepressant drugs on the periodontium in clinical and preclinical models.

Fluoxetine (an SSRIs) reduced periodontal progression [77], alveolar bone loss and inflammatory responses [79] in rats with induced periodontitis. Furthermore, it appears to influence periodontal genesis in mice without any clinically relevant effects by decreasing the number of periodontal cells [11]. The beneficial effects of fluoxetine could be explained by its ability to reduce pathological inflammatory response and gene transcription when administered in an animal model [60,83]. Intake of tianeptine, imipramine and desipramine is associated with a reduction in the level of alveolar bone loss and periodontal inflammation in mice. These molecules appear to exhibit immunomodulatory effects and can modulate bone remodelling and key inflammatory mediators [72,74,81,82]. Desipramine (belonging to a tricyclic antidepressant) also appears to attenuate alveolar bone loss and modulate gene expression in mice [72].

In light of the results that emerged on animal models, although fluoxetine, imipramine, tianeptine, desvenlafaxine and desipramine appear to have anti-inflammatory and immunomodulatory properties, it is not possible to state with certainty the mechanism of action with which these drugs intervene at the periodontal level [71,72,73,81,82].

The results of studies performed on clinical models are contradictory. The recent study by Hakam et al. analysed the records of 582 patients divided into those with and without periodontitis [66]. The periodontal parameters of patients taking different classes of antidepressants were compared with those of non-users. Subjects taking antidepressants (especially SSRIs such as fluoxetine or mixed classes) had statistically higher rates of alveolar bone loss and clinical attachment loss than periodontal patients not taking these drugs. Therefore, there are conflicting opinions on the effect of antidepressants on periodontal health: indeed, studies have shown improvements in periodontal and immunological indices with desvenlafaxine and fluoxetine treatment in patients with periodontitis and depression, while others have highlighted how the use of some antidepressants in the presence of periodontitis and depression limits periodontal loss and influences periodontal epidemiological indices [75], such as bleeding on probing and alveolar bone loss [78].

The antidepressant that appears to be more damaging to the periodontium is venlafaxine, which worsens PPD, CAL, GI and DI [67,68] and exacerbates bone loss due to synaptic inhibition of serotonin uptake, which has negative effects on the skeleton [76].

The contrasting results may be due to the different categories of antidepressants used in the studies included in this review: probably the negative or positive effects are linked precisely to the category of the drug and its application.

This review, which would seem to show that there is no effect on periodontal health in patients taking antidepressants (category), shows a strong risk in the studies—animal or human studies, different sample numbers, different antidepressants used and different dosages, different epidemiological indices analysed—these data could confuse the results emerging from this review, but also explain the reason for their diversity.

For future studies, it would be useful to use a uniform sample, with the same degree of periodontitis (even if induced), to which is administered the same type and dosage of antidepressant. In addition, it is possible to make a comparison with an untreated control group or with other groups of patients treated with other antidepressants, in an attempt to highlight the side effects that may arise.

## 5. Conclusions

Depressive disorders and periodontal disease have been reported to share distinct behavioural, inflammatory and bacterial translocation mechanisms. In particular, the pharmacology of depressive disorders and their association with the course of periodontal disease have been analysed in clinical and preclinical models.

Our analysis suggests that in the presence of comorbidity between periodontitis and depression, pharmacological treatment with SNRIs, SSRIs and mixed antidepressants is associated with an improvement in periodontal parameters (such as probing pocket depth, bleeding on probing, clinical attachment loss and bone loss), with the exception of venlafaxine (with worsening of periodontal indices, including bone loss). Therefore, it is not possible to provide a clear idea of the effect of antidepressants on periodontal health, which depends on their type and dosage.

Healthcare professionals (especially oral and mental health professionals) should assess the adherence to medication in patients with a history of periodontitis and depression.

Pharmacological treatment may modulate and reduce the inflammatory burden in patients diagnosed with both periodontitis and depression. Further studies are needed to analyse the cause-and-effect mechanisms underlying the depression–periodontitis associations and to investigate the anti-inflammatory and immunomodulatory effects of different antidepressants on the periodontium, using a uniform sample of patients with periodontal disease and the same class of antidepressant. These effects have not been well studied.

## Figures and Tables

**Table 1:** Results of studies [66,67,68,70,71,72,73,74,75,76,77,78,79,80,81,82,83].

Study	Study Design and Population	Presence of Periodontitis/Depression at t0	Comparison Groups and Pharmacological Administration	Tested Parameters/Analyses Conducted	Outcome
Hakam et al., 2022 [66]	Retrospective study conducted on humans	Presence of periodontitis; no diagnosis of depression (users and non-users of antidepressants)	Group 1 (user group): subjects who use antidepressants; Group 2 (non-user group): subjects who do not use antidepressants.	Variables obtained: type of antidepressant, age, sex, smoking, mild systemic diseases, CAL, BL	Antidepressant use was associated with significantly better BL and CAL in patients with periodontitis. Analysing pharmacological classes separately, SSRI (selective serotonin reuptake inhibitor) users and users of multiple pharmacological classes had lower BL and CAL than non-users.
Bey et al., 2020 [67]	Case–control study on humans	Absence of periodontitis/presence of depression at t0	Group 1: control group diagnosed as depressed at the first visit; Group 2: depressed patients taking fluoxetine 20 mg/day; Group 3: patients taking venlafaxine 75 mg/day.	DI, CI, PPD, CAL	Fluoxetine and venlafaxine are associated with a worsening of periodontal parameters, when compared with the group not taking antidepressant drugs.
Majeed et al., 2024 [68]	Case–control study conducted on humans	Absence of periodontitis/diagnosis of depression at t0	Group 1: Subjects identified by a psychiatrist as having a mental illness (control) after presenting to the psychiatry outpatient department (OPD); Group 2: patients who are taking venlafaxine; Group 3: patients who are taking fluoxetine.	CAL, PPD, CI, DI	Antidepressants can be a risk factor for periodontal health, with an increase in periodontal parameters, as these drugs can put periodontal tissues at risk.
Hasan et al., 2019 [70]	RCT on humans	Periodontitis at t0/no depression	Group 1 (control group): standard periodontal therapy; Group 2: periodontal therapy + amitriptyline gel; Group 3: periodontal therapy + amitriptyline mouthwash.	PD, AL, tooth mobility; PI, GI, BOP; saliva sample collection and estimation of TNF-α, PGE2 and NO	Improvement in periodontal parameters in the amitriptyline + gel/mouthwash group compared to the 1 standard therapy group.
Hassan et al., 2022 [71]	Experimental study on rats	No periodontitis/no depression at t0	Control group: distilled water; Test group: 10 mg per day per kg of amitriptyline.	Radiographic analysis (CBCT), histomorphometric analysis, anti-OPN and H&E immunohistochemical staining	Amitriptyline worsened periodontal destruction and increased the expression of anti-OPN in periodontal tissues, reducing bone mineral density.
Branco-de-Almeida et al., 2020 [72]	Experimental study on rats	Induced periodontitis/no depression at t0	Group 1 (control group): rats without ligation (saline); Group 2 (ligation group): rats with induced periodontitis treated with saline solution; Group 3 (ligation + desipramine group): rats with ligation-induced periodontitis treated with desipramine (20 mg/kg/day).	RNA isolation and gene expression of IL-1β, iNOS, COX-2, MMP-9 and TIMP-1; zymography to evaluate MMP-9 activity	Desipramine reduced alveolar bone loss by modulating gene expression of inflammatory markers.
Li et al., 2022 [73]	Experimental study on rats	Induced periodontitis/no depression at t0	Group 1: control group; Group 2: control group with aSMase (acid sphingomyelinase) inhibition; Group 3: periodontitis group; Group 4: periodontitis group with aSMase (acid sphingomyelinase) inhibition; Group 5: MetS group (metabolic syndrome); Group 6: MetS group (metabolic syndrome) with aSMase (acid sphingomyelinase) inhibition; Group 7: periodontitis and MetS (metabolic syndrome) group; Group 8: periodontitis and MetS (metabolic syndrome) group with aSMase (acid sphingomyelinase) inhibition.	Metabolic measurements, micro-computed tomography and bone volume fraction analysis, acid phosphatase staining, histological tissue processing and pathological evaluation, cell cultures to evaluate alveolar bone loss, osteoclast formation, periodontal inflammation and pro-inflammatory gene expression	Imipramine inhibited the synergy between metabolic syndrome (MetS) and periodontitis on alveolar bone loss, proposing acid sphingomyelinase (aSMase) as a therapeutic target of periodontitis exacerbated by MetS.
Yamawaki et al., 2022 [74]	Experimental study on rats	Induction of LPS-PG/no depression at t0	Active group treated with imipramine (20 mg/kg) 1 h before LPS-PG (lipopolysaccharide from porphyromonas gingivalis) injection (5 mg/kg).	Cell culture and cell immunoreactivity assay using electrochemiluminescence (ECL) reagent	Imipramine is associated with a reduction in the expression of TNF (tumour necrosis factor) and Il-1 (interleukin 1) in the hippocampus, 24 h after introduction; furthermore, it attenuated microglial-induced neuronal death by inhibiting signaling of an inflammation factor in microglia (NF-κB).
Bhatia et al., 2018 [75]	Observational study on humans	Presence of chronic periodontitis/presence of depression at t0	Group 1 (test): they took a daily dose of 50 mg/day of desvenlafaxine; Group 2 (control group): diagnosed with depression at the first visit who had not started any antidepressant medication.	PI, GI, SBI, BOP, PPD, AL	Patients treated with desvenlafaxine had shallower pocket depth and less bleeding in survey.
Carvalho et al., 2010 [76]	Experimental study on rats	Experimentally induced periodontitis/absence of depression at t0	Group 1: sham-operated (SO); Group 2: experimental periodontitis treated with vehicle; Groups 3 and 4: rats without induced periodontitis treated with 10 or 50 mg/kg of venlafaxine; Groups 5 and 6: rats with induced periodontitis treated orally with venlafaxine 10 or 50 mg/kg.	Bone loss analysed morphometrically and histopathological and immunohistochemical analysis for TNF-α and iNOS	High-dose venlafaxine (50 mg/kg) increased bone loss and worsened the inflammation condition of the tested animals. Furthermore, the drug increased the immunoreactivity of inflammatory biomarkers such as TNF (tumour necrosis factor).
Aguiar et al., 2013 [77]	Experimental study on rats	Experimentally induced periodontitis (before depression)/experimentally induced depression at t0	Groups 1: non-stressed rats; Group 2: non-stressed rats + daily fluoxetine (20 mg/kg) Group C: stressed rats; Group 3: stressed rats + daily fluoxetine (20 mg/kg).	Histological analyses and immunohistochemical staining for IL-1β and IL-6	Animal models with depression and periodontitis had greater bone loss than the non-depressed periodontitis group. Furthermore, fluoxetine reduced levels of bone loss in animal models of induced periodontitis and stress-induced depression.
Bhatia et al., 2015 [78]	Cross- sectional observational study on humans	Periodontitis present/and depression diagnosed at the first visit at t0	Group 1 (test group): periodontal patients taking 20 mg/day of fluoxetine for at least 2 months; Group 2 (control group): periodontal patients who had yet to start antidepressant treatment.	PI, GI, SBI, BOP, PPD, AL	Except plaque index (PI), all parameters were lower in group taking fluoxetine compared to the control group (depressed patients with periodontitis).
Branco-de-Almeida et al., 2012 [79]	Experimental study on rats	Experimentally induced periodontitis/depression absent at t0	Group 1: control rats (without ligation); Group 2: rats with ligation + placebo (saline); Group 3: ligation rats + fluoxetine (20 mg/kg/day).	Bone loss by histometric assessment, expression of IL-1β, COX-2, MMP-9 and iNOS and MMP-9 activity	The periodontitis group and fluoxetine demonstrated less alveolar bone loss at histometric evaluation compared to the group with induced periodontitis alone and placebo. Furthermore, in the fluoxetine group there was a reduced inflammatory expression of IL-1β (interleukin 1-beta) and COX-2 (cyclooxygenase-2).
Regueira et al., 2017 [80]	Experimental study on rats	Absence of periodontitis/depression at t0	Group 1: sodium chloride administered throughout the pregnancy; Group 2: sodium chloride administered throughout pregnancy and breastfeeding; Group 3: fluoxetine administered throughout the pregnancy; Group 4: fluoxetine administered throughout pregnancy and breastfeeding.	Histometrical, histochemical and immunohistochemical analysis of the maxillary first molar periodontium region of rat pups made under light microscopy; periodontal ligament collagen qualitatively evaluated under a polarizing light microscope	Decreases in osteoblasts, fibroblasts and mercatoblasts were observed, but only in the group in which fluoxetine was taken until the breastfeeding period. However, it is not possible to determine whether this cellular deficiency actually influenced periodontogenesis, as the morphological descriptive analysis did not highlight any alterations or structural elements evident in the periodontal conformation.
Breivik et al., 2006 [81]	Experimental study on rats	Experimentally induced periodontitis (before depression)/experimentally induced depression at t0	Experiment 1: Group 1: mice with periodontitis and induced depression (OB); Group 2: mice with induced periodontitis without depression. Experiment 2: Group 1: OB rats treated with tianeptine; Group 2: OB mice treated with saline; Group 3: control mice.	Radiographic bone loss, analysis of serum corticosterone, tumour necrosis factor TNF-α, IL-10 and TGF-β; RNA isolation in the hippocampus	Depressed mice (OB) had a higher susceptibility to periodontitis than healthy controls without induced depression. Tianeptine treatment of OB rats significantly inhibited periodontal bone loss, normalised behavioural responses, increased TGF-1β levels and abolished the decrease in TNF-α, but did not attenuate the increase in corticosterone response and decreased hippocampal GR expression.
Breivik et al., 2006 [82]	Experimental study on rats	Induced periodontitis/depression absent at t0	Experiment 1: Group 1 (active group): dexamethasone; Group 2 (control group): physiological solution. Experiment 2: Group 1 (active group): 10 mg/kg per day of tianeptine; Group 2 (control group): physiological solution.	Radiographic bone loss, analysis of serum corticosterone, tumour necrosis factor TNF-α, IL-10 and TGF-β; RNA isolation in the hippocampus	Tianeptine-treated group showed significantly reduced periodontal bone loss, increased plasma levels of TNF-α and transforming growth factor-1β; no significant difference was found in corticosterone levels.
Muniz et al., 2018 [83]	Systematic review of 5 experimental studies on rats	Studies involved: Breivick et al., 2006 [81]; Breivick et al., 2006 [82]; Carvalho et al., 2010 [76]; Branco-de-Almeida et al., 2012 [79]; Aguiar et al., 2013 [77];	/	Parameters extracted from each selected study: author, country, number of animals involved, antidepressant used, use of ligature (yes or no), intervention (if any) in the control group, number of days with the ligature placed, number of days the antidepressant was administered, measurement of ABL in each experimental group and additional information.	With the exception of venlafaxine, the antidepressant treatments studied (tianeptine and fluoxetine) can modify the reactivity of the stress response system and modulate susceptibility to periodontitis.

Abbreviations. PPD: Probing pocket depth; CAL: Clinical attachment loss; AL: Attachment loss; BOP: Bleeding on probing; GI: Gingival index; BL: Bone level; PI: Plaque index; SBI: Sulcular bleeding index; DI: Debris index; CI: Calculus index; TNF-α: Tumour necrosis factor; PGE2: Prostaglandin E2; IL: Interleukin; iNOS: Inducible nitric oxide synthase; COX-2: Cyclooxygenase-2; MMP-9: Matrix metallopeptidase 9; TIMP-1: Tissue inhibitor of metalloproteinase 1; TGF-β: Transforming growth factor beta; OPN: Anti-osteopontin antibody; H&E: Haematoxylin plus eosin.

**Table 2:** Antidepressants and their effects on the periodontium.

Article	Antidepressant Used	Effects on Periodontium
Hakam et al., 2022 [66]	SSRI (selective serotonin reuptake inhibitors), SNRI (serotonin-norepinephrine reuptake inhibitors), tricyclic, atypical and MAO (monoamine oxidase inhibitors) categories	Antidepressants improve BL and CAL
Bey et al., 2020 [67]	Fluoxetine and Venlafaxine	Fluoxetine and Venlafaxine worsen periodontal health
Majeed et al., 2024 [68]	Fluoxetine and Venlafaxine	Fluoxetine and Venlafaxine worsen periodontal indices
Hasan et al., 2019 [70]	Amitryptiline	Amitryptiline improves periodontal health and periodontal indices
Hassan et al., 2022 [71]	Amitryptiline	Amitryptiline worsens periodontal health
Branco-de-Almeida et al., 2020 [72]	Desipramine	Desipramine reduces alveolar bone loss
Li et al., 2022 [73]	Imipramine	Imipramine improves periodontal health
Yamawaki et al., 2022 [74]	Imipramine	Imipramine inhibits LPS-PG-induced inflammatory responses in microglia and improves periodontal disease-related neural damage
Bhatia et al., 2018 [75]	Desvenlafaxine	Desvenlafaxine improves PPD and BOP
Carvalho et al., 2010 [76]	Venlafaxine	Venlafaxine increases inflammation and bone loss
Aguiar et al., 2013 [77]	Fluoxetine	Fluoxetine reduces bone loss
Bhatia et al., 2015 [78]	Fluoxetine	Fluoxetine improves GI, SBI, BOP, CAL (but not plaque index)
Branco-de-Almeida et al., 2012 [79]	Fluoxetine	Fluoxetine reduces inflammation and bone loss
Regueira et al., 2017 [80]	Fluoxetine	Periodontal tissue may be sensitive to fluoxetine, and its interference in reducing periodontal cells depends on exposure time during lactation
Breivik et al., 2006 [81]	Tianeptine	Tianeptine inhibits bone loss
Breivik et al., 2006 [82]	Tianeptine	Tianeptine inhibits bone loss
Muniz et al., 2018 [83]	Fluoxetine, Tianeptine and Venlafaxine	Only Venlafaxine study was not to able to find any significant alveolar bone loss reduction, while others showed positive effects

Abbreviations. PPD: Probing pocket depth; CAL: Clinical attachment loss; AL: Attachment loss; BOP: Bleeding on probing; GI: Gingival index; BL: Bone level; PI: Plaque index; SBI: Sulcular bleeding index.

**Table 3:** Risk of bias of articles [66,67,68,70,71,72,73,74,75,76,77,78,79,80,81,82,83].

	Adequate Sequence Generated	Allocation Concealment	Blinding	Incomplete Outcome Data	Registration Outcome Data
Hakam et al., 2022 [66]	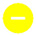	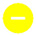	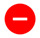	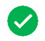	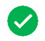
Bey et al., 2020 [67]	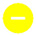	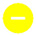	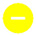	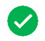	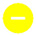
Majeed et al., 2024 [68]	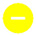	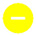	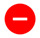	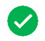	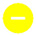
Hasan et al., 2019 [70]	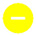	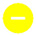	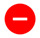	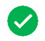	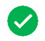
Hassan et al., 2022 [71]	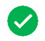	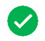	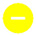	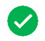	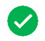
Branco-de-Almeida et al., 2020 [72]	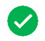	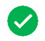	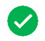	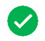	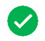
Li et al., 2022 [73]	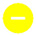	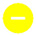	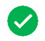	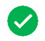	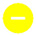
Yamawaki et al., 2022 [74]	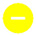	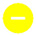	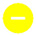	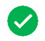	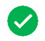
Bhatia et al., 2018 [75]	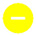	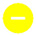	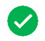	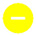	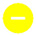
Carvalho et al., 2010 [76]	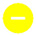	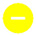	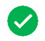	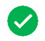	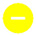
Aguiar et al., 2013 [77]	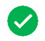	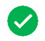	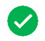	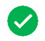	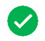
Bhatia et al., 2015 [78]	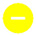	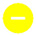	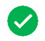	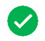	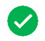
Branco-de-Almeida et al., 2012 [79]	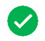	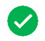	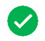	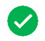	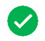
Regueira et al., 2017 [80]	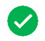	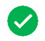	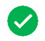	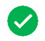	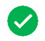
Breivik et al., 2006 [81]	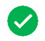	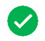	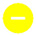	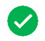	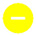
Breivik et al., 2006 [82]	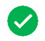	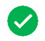	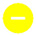	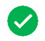	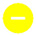
Muniz et al., 2018 [83]	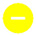	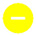	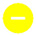	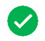	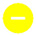
